# Mitochondria: a new therapeutic target in chronic kidney disease

**DOI:** 10.1186/s12986-015-0044-z

**Published:** 2015-11-25

**Authors:** Simona Granata, Alessandra Dalla Gassa, Paola Tomei, Antonio Lupo, Gianluigi Zaza

**Affiliations:** Renal Unit, Department of Medicine, University-Hospital of Verona, Piazzale A. Stefani 1, 37126 Verona, VR Italy

**Keywords:** Mitochondria, Oxidative stress, Chronic kidney disease, Antioxidant drugs, Natural plants extracts, Drugs

## Abstract

Cellular metabolic changes during chronic kidney disease (CKD) may induce higher production of oxygen radicals that play a significant role in the progression of renal damage and in the onset of important comorbidities. This condition seems to be in part related to dysfunctional mitochondria that cause an increased electron “leakage” from the respiratory chain during oxidative phosphorylation with a consequent generation of reactive oxygen species (ROS).

ROS are highly active molecules that may oxidize proteins, lipids and nucleic acids with a consequent damage of cells and tissues.

To mitigate this mitochondria-related functional impairment, a variety of agents (including endogenous and food derived antioxidants, natural plants extracts, mitochondria-targeted molecules) combined with conventional therapies could be employed.

However, although the anti-oxidant properties of these substances are well known, their use in clinical practice has been only partially investigated. Additionally, for their correct utilization is extremely important to understand their effects, to identify the correct target of intervention and to minimize adverse effects.

Therefore, in this manuscript, we reviewed the characteristics of the available mitochondria-targeted anti-oxidant compounds that could be employed routinely in our nephrology, internal medicine and renal transplant centers. Nevertheless, large clinical trials are needed to provide more definitive information about their use and to assess their overall efficacy or toxicity.

## Background

### Chronic Kidney Disese (Ckd)

During CKD, the progressive deterioration of renal function [1] induces several biological and clinical dysfunctions including alteration in cellular energetic metabolism, change in nitrogen input/output, protein malnutrition, resistance to insulin and considerable enhancement of synthesis of inflammation/oxidative stress mediators [2–6].

Several authors have reported that in CKD, even in the early stage, there is an abundant production of reactive oxygen species (ROS) [7] mainly due to an hyperactivation of the nicotinamide adenine dinucleotide phosphate (NADPH) oxidase [8–10], elevated synthesis of oxidative stress markers [e.g., F2-isoprostanes, malondialdehyde (MDA), advanced oxidation protein products (AOPP)] and release of uremic toxins. The level of all these factors is inversely correlated with the glomerular filtration rate (GFR) [5, 11–13].

The early stages of CKD require nutritional and pharmacological interventions to minimize uremic symptoms and maintain volume homeostasis (conservative therapy), while in the final stage of renal failure these alterations may induce the development of severe and life-threatening clinical complications and renal replacement therapies (RRT: hemodialysis and peritoneal dialysis) are necessary.

Although necessary to ensure patients’ survival, hemodialysis (HD) and peritoneal dialysis (PD) exacerbate oxidative stress [14, 15] by exposing blood to the contact with low biocompatible dialytic devices or fluids. In HD, the contact of peripheral blood mononuclear cells (PBMCs) with plastificants and filters [16] and the microbial contamination together with the release of pyrogens in dialysate induce ROS synthesis as part of the immune response [17–22]. Moreover, similarly to CKD, HD patients show an increased free radical-catalyzed peroxidation of arachidonoyl lipids with elevated production of lipid peroxidation products [MDA, 4-hydroxynonenal (HNE) and F2-isoprostanes] [23, 24]. Other markers of oxidative stress shown to be elevated in HD include lipid hydroperoxides, oxidized-LDL and AOPP [11, 25–30].

At the same time, plasma levels of both non-enzymatic (e.g., vitamin C, vitamin E) [31] and enzymatic antioxidants [e.g., superoxide dismutase (SOD) and catalase, glutathione peroxidase (GPx) and paraoxonase (PON1)] are reduced in CKD and HD patients [32–35].

The above mentioned imbalance between oxidants and antioxidants in patients with advanced renal impairment can accelerate renal injury progression and may contribute to the high rate of clinical complications in both CKD patients in conservative and dialysis treatment. Major complications include cardiovascular disease, atherosclerosis, amyloidosis and DNA-Damage-Associated Malignancy [36].

Additionally, oxidative stress together with altered nutritional status, inflammation and cardiovascular disease may determine the onset and development of that condition known as “MIA syndrome” described by Stenvinkel et al. [37].

Similarly, during peritoneal dialysis (PD) treatment, “unphysiological” fluids characterized by high lactate and glucose concentration, high osmolality and glucose degradation products (GDPs) [38] could determine local and systemic oxidative stress. The latter may be aggravated by chronic inflammation, diabetes, advanced age, and loss of antioxidants such as vitamins C and E [39, 40].

Finally, it is unquestionable that oxidative stress is an important cofactor contributing also to immune system deregulation [41].

### Mitochondria and Ckd

Mitochondria participate in numerous cellular functions including ion homeostasis, heme and steroid synthesis, calcium signaling, apoptosis [42–45]. The prominent role of this organelle is to generate energy for cellular metabolism by the oxidative phosphorylation system (OXPHOS).

Electrons derived from cellular metabolism reach the mitochondria through two coenzymes, nicotinamide adenine dinucleotide (NADH)- and flavin adenine dinucleotide (FADH2). Then they undergo a passage throughout the electron transport chain that consists of five protein complexes located in the inner mitochondrial membrane.

**Fig. 1:**
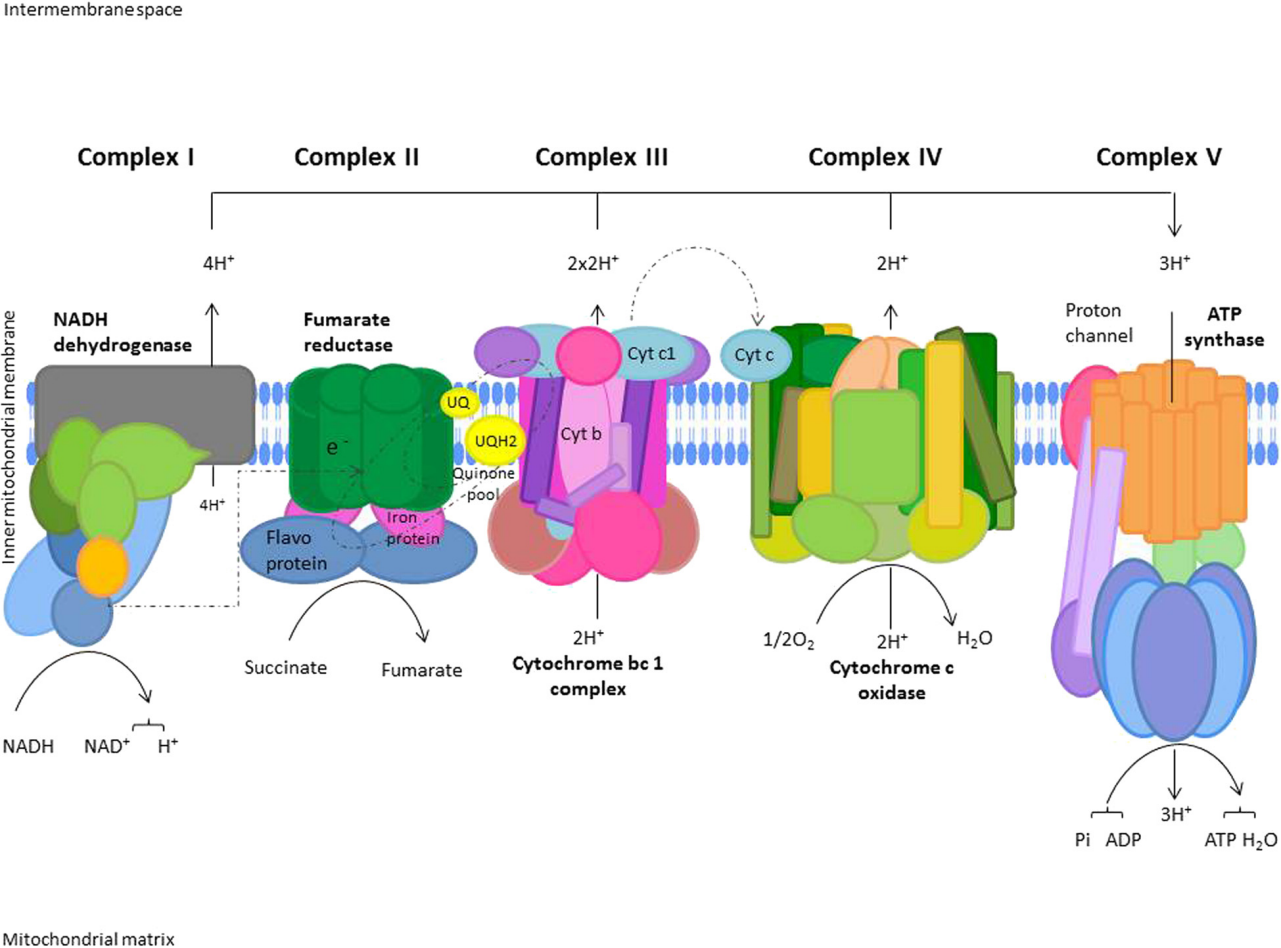
Oxidative Phosphorylation System (OXPHOS). Electrons derived from cellular metabolism reach complex I or complex II through NADH or FADH2, respectively. These electrons are then transferred to coenzyme Q (ubiquinone), a carrier of electrons from complex I or II, to III. In the latter, particles are shifted form cytochrome b to cytochrome c with a consequent transfer to Complex IV (cytochrome oxidase) where they reduce O2. This electrons transport through mitochondrial complexes is coupled to shipment of protons in the intermembrane space. The electrochemical gradient generated is used by Complex V for ATP synthesis. Adapted from the KEGG Oxidative phosphorylation pathway (Reference number: 00190, http://www.genome.jp/kegg-bin/show_pathway?map00190)

Electrons pass through complexes I, III and IV thanks to a proton gradient generated by the transport of these particles to the outer side of the inner mitochondrial membrane. Complex V then translates energy derived from electron transport to ATP synthesis [46] (Fig. 1).

In this process, the electron leakage from the respiratory chain induces the conversion of oxygen (0.4–4 %) in superoxide radicals [47]. As a consequence mitochondria are the primary source of ROS.

**Fig. 2:**
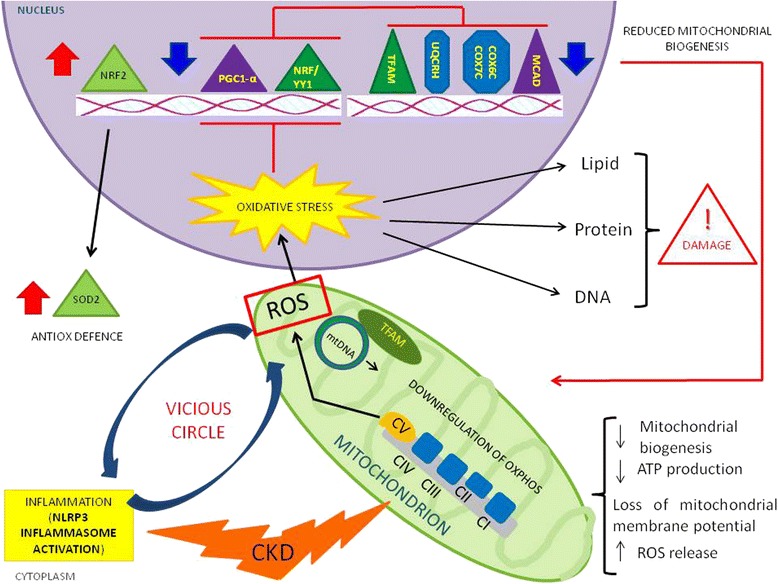
Schematic representation of the mitochondrial involvement in chronic kidney disease (CKD). In this pathological condition, mitochondrial impairment (mainly characterized by a reduction in mitochondrial biogenesis, loss of mitochondrial membrane potential, and drop of ATP production) causes a great release of ROS that could contribute to chronic microinflammation through NLRP3 inflammasome activation. At the same time, during CKD, nuclear factor erythroid 2-related factor 2 (NRF-2) and one of its target gene superoxide dismutase 2 (SOD2) are up-regulated by oxidative stress, in the attempt to neutralize ROS production. Notably, this effect has been observed by our group [54] in PBMCs

Recent findings emphasize the involvement of mitochondria in progression of chronic kidney damage [48, 49] (Fig. 2) particularly due to a reduction in mitochondrial DNA (mtDNA) copy number, loss of mitochondrial membrane potential (Δψm), and drop of ATP production [50]. Mitochondria are also involved in apoptosis and epithelial to mesenchymal transition of renal tubular epithelial cells contributing to the fibrogenic process [51].

Our group has recently demonstrated that the activity of Complex IV (a key regulator of respiratory chain activity) is reduced in PBMC of CKD/HD patients [49]. This causes a drop in ATP production and exacerbates oxidative stress because these dysfunctional organelles release a great amount of ROS. Interestingly the mitochondrial ROS are able to activate NLRP3 inflammasome and thus contribute to CKD-related chronic microinflammation [52].

This mitochondria-induced NLRP3 inflammasome activation has been also reported by other groups in animal model of proteinuria-induced renal tubular injury [53].

We also showed [54] a deregulated mitochondrial-related intracellular machinery in uremic patients treated with peritoneal dialysis (PD). A group of genes encoding for mitochondrial biogenesis (PGC-1α, NRF1 and TFAM) and functional proteins (COX6C, COX7C, UQCRH and MCAD) were down-regulated in PD compared to healthy subjects.

At once, nuclear factor erythroid 2-related factor 2 (NRF-2) and one of its target gene superoxide dismutase 2 (SOD2) were up-regulated in peritoneal dialysis-treated patients in the attempt to neutralize ROS over-production.

However, whether these mitochondrial abnormalities represent a causative factor or an outcome of cellular injury during this process remains to be investigated.

### Mitocondrial-induced oxidative stress: a New therapeutic target in Ckd

Mitochondria could be in future a valuable pharmacological target for patients with renal impairment and a variety of agents, combined with conventional therapies and an appropriate life style, targeting mitochondria-derived oxidative stress, could prevent and slow-down the progression of CKD and minimize the development of severe systemic complications.

Nevertheless, although the anti-oxidant properties of most of these agents are well known, their use in clinical nephrology are only partially investigated.

**Fig. 3:**
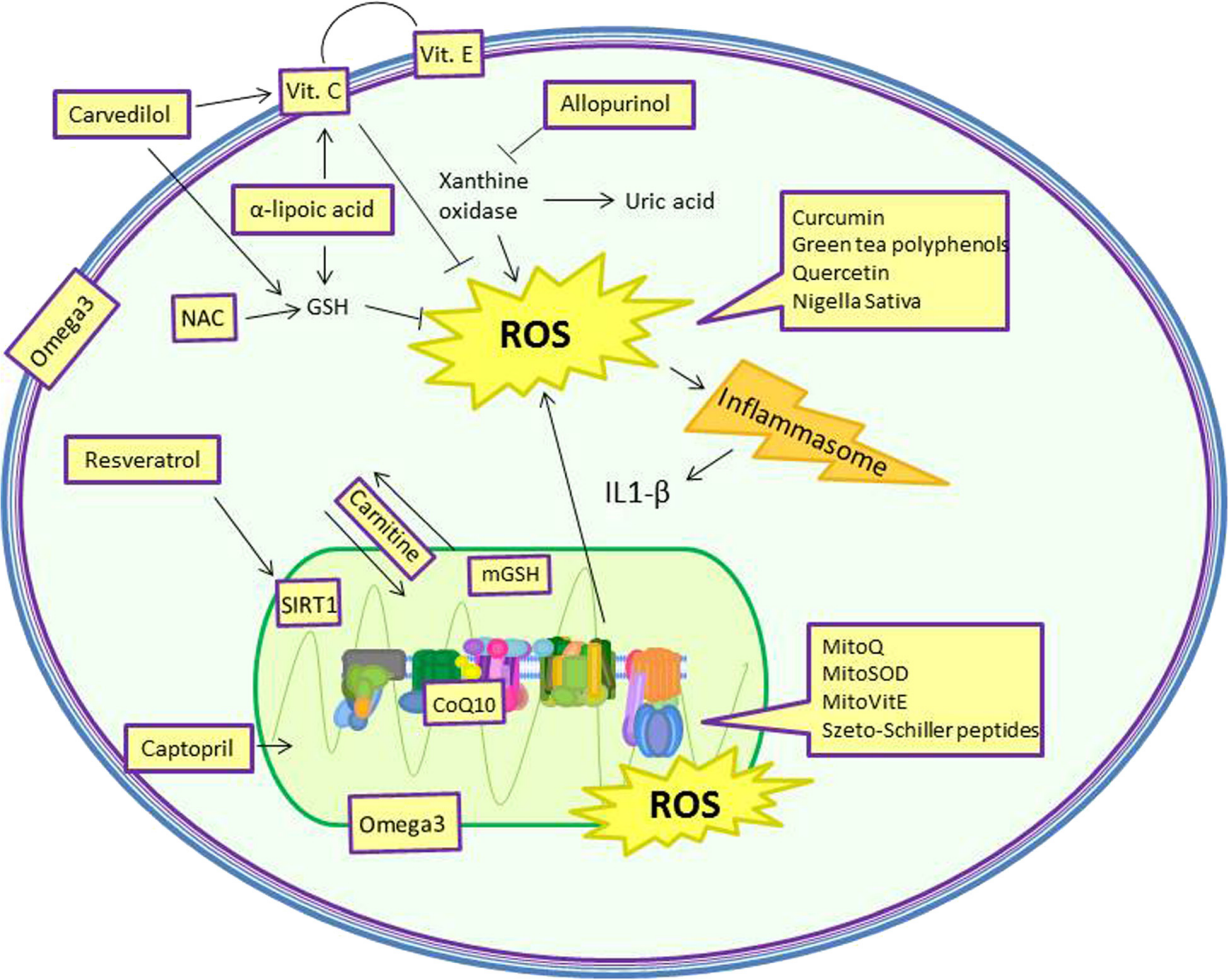
Target sites of major anti-oxidants agents

At the moment, the available mitochondria-targeted and anti-oxidant agents are (Fig. 3):

1. Endogenous and food derived antioxidants;
2. Natural plants extracts;
3. Conventional drugs with favorable antioxidant side effects;
4. Mitochondria-targeted molecules.

## Endogenous and food derived antioxidants

### L-Carnitine

L-carnitine (4-N-trimethylammonium-3-hydroxybutyric acid) mainly derives from diet (75 %) with a bioavailability that ranges from 54-72 % and it is synthesized endogenously (primarily in liver and kidney) from two essential amino acids: lysine and methionine [55].

It mediates the transport of fatty acids across the mitochondrial inner membrane from the cytosol to the mitochondrial matrix for their β-oxidation. This leads to acetyl coenzyme A production that, entering tricarboxylic acid cycle, improves mitochondrial respiratory chain activity and reduces ROS formation [56].

Additionally, L-carnitine is able to directly reduce free radical generation by scavenging ROS and chelating iron [57] and it may act as secondary antioxidant by increasing the production/activity of antioxidant enzymes and by inhibiting lipid peroxidation and xanthine oxidase activity [58, 59].

As demonstrated in animal model, L-carnitine reduces MDA content and restores glutathione (GSH) levels in aorta, heart and kidney tissues [60].

Patients with CKD in conservative therapy have higher plasma concentrations of L-carnitine than healthy individuals [61–64]. In contrast, a large number of studies have reported low plasma and muscle L-carnitine levels in CKD patients undergoing chronic hemodialysis [65–67] correlated with dialysis vintage [61–64, 68, 69]. This is mainly due to the efficient removal of the compound during the treatment together with a reduction in L-carnitine dietary intake and endogenous synthesis [65, 67, 70].

The depletion in L-carnitine is associated with important clinical problems and symptoms, most notable of which are anemia hyporesponsive to erythropoietin therapy, intradialytic hypotension, cardiomyopathy and skeletal muscle dysfunction manifested as generalized fatigability [71].

Pertosa et al. [72], also, reported that 3 months supplementation of L-carnitine was also able to reduce intracellular levels of phosphorylated proteins and jun-N-terminal Kinase (JNK) activity in PBMC from HD patients treated with cellulosic membrane. This treatment caused a significant improvement of cellular defense against chronic inflammation and oxidative stress, most likely by modulating the specific signal transduction cascade activated by an overproduction of proinflammatory cytokines and oxidative stress.

Therefore, in the last years, the L-Carnitine supplementation in HD has been emphasized and several nephrology groups have started clinical research programs and trials. However, the results of most of these studies resulted unconvincing and conflicting. This could be due to the small population employed, the short duration of follow-up and the absence of a correct selection or adjustment for clinical manifestations [73–78].

Based on these clinical evidences, expert consensus groups and federal agencies have recommended L-Carnitine not for routine use, but for dialysis patients with specific indications. In 1999, the FDA approved intravenous L-Carnitine for use in dialysis-related carnitine deficiency, as defined by low L-Carnitine levels [79].

Subsequently, expert consensus panels of the American Association of Kidney Patients and of the National Kidney Foundation have recommended intravenous L-Carnitine for treatment of erythropoietin-resistant anemia, dialysis hypotension, cardiomiophaty and muscle weakness [80]. Use of oral L-Carnitine was discouraged because of limited bioavailability, scarcity of supportive studies, and formation of toxic metabolities via intestinal metabolism.

### Coenzyme Q10 (CoQ10)

CoQ10 is a biological element belonging to the mitochondrial electron transport chain that moves electrons from complex I/II to complex III [81] endogenously synthesized from tyrosine in several human tissues or introduced with diet (meat, fish, nuts, and some oils) [82].

Because its chemical characteristics (high molecular weight, strong lipophily, and weak solubility in water solution) CoQ10 has poor bioavailability in humans [83].

It prevents membrane lipid peroxidation, apoptosis by inhibiting permeability transition pore (PTP) opening and mitochondrial membrane potential depolarization, and it is required for the uncoupling proteins function [84–86]. CoQ10 improves the oxygen consuming, ATP production and mitochondrial protein synthesis [87].

Moreover, CoQ10 is capable of recycling and regenerating other antioxidants such as tocopherol and ascorbate [88, 89]. All these characteristics determine its clinical effects. In particular, CoQ10 may exerts important cardiovascular protective properties in patients affected by renal failure.

Atherosclerotic cardiovascular disease (CVD) is the main cause of high mortality rates among patients with advanced CKD [90]. This high incidence of cardiovascular (CV) death rate is 5–20 times higher in these patients population compared with those with normal renal function age and sex-matched and seems to be primarily associated with non-traditional risk factors as oxidative stress [91, 92].

Additionally, epicardial fat tissue (EFT), the visceral adipose tissue surrounding the subepicardial coronary vessels has recently been recognized as a new risk factor for atherosclerotic heart disease in PD and HD patients [93, 94]. EFT is a lipid-storing depot [95, 96] and secretes proatherosclerotic and proinflammatory cytokines [97–99].

Macunluoglu et al., in accordance with previous studies [91, 100] demonstrated that Co-Q10 levels were significantly decreased in HD patients compared to healthy controls and inversely correlated with EFT thickness [101].

Authors hypothesized that increased EFT in HD patients can be another source of pro-inflammatory cytokines and pro-oxidant molecules which cause the consumption of Co-Q10 as antioxidant molecule. At the same time, the increased oxidative stress due to dialysis membranes, dialysate water and extracorporeal blood circulation could cause EFT overproduction as a part of the atherosclerotic process.

In another study it has been reported that Co-Q10 supplementation for 6 months reduces oxidative stress in HD patients but it was unclear whether this benefit would be translated into good clinical outcomes [100].

In an animal model Ishikawa et al. demonstrated that heminephrectomized rats fed with a CoQ10-supplemented diet showed lower levels of ROS and better renal function [102]. However, no clinical data strongly support this finding.

Moreover in mouse models of type 2 diabetes, CoQ10 introduction reduced oxygen consumption, mitochondrial fragmentation glomerular hyperfiltration and proteinuria [103, 104].

However, although these encouraging results, CoQ10 effects on CKD remain to be determined by additional *in vivo* studies and clinical trials.

### Alpha-lipoic acid (ALA)

ALA is commonly found in vegetables (e.g., spinach, broccoli, tomato) and meat, but it can be also enzymatically synthesized by octanoic acid and cysteine in human mitochondria.

Because of its amphipathic structure is ubiquitously distributed in several cellular structures and in mitochondria where it acts as a cofactor for pyruvate dehydrogenase and α-keto-glutarate dehydrogenase complexes.

Its neutralizes several free radicals [105], reduces the oxidized form of vitamin C and GSH, prevents the synthesis of free radicals by forming stable complex with the catalyzers Mn^2+^, Cu^2+^, and Zn^2+^ and chelating Fe^2+^ [106] and limits the inflammation through the inhibition of NFkB [107]. It is an inducer of NRF2-mediated antioxidant gene expression and activates PPAR-α and –ɣ regulating the expression of several enzymes regulators of glucose and lipid metabolism [108].

Kim et al. reported, also, that ALA decreases vascular calcification by reducing vascular smooth muscle cells (VSMC) apoptosis by preserving anti-oxidant mitochondrial functions and activating Akt [109].

All these biochemical effects confer to this cofactor important antioxidant properties [110] that can be exploited in CKD patients to slow down the progression of renal damage and to control the onset of severe cardiovascular complications.

However, only few papers have described the clinical impact of ALA supplementation in CKD with contrasting results.

In a recent paper, HD patients receiving a daily dose of ALA (600 mg) for 8 weeks reported only a reduction in C-reactive protein (CRP) level, which is a risk factor for cardiovascular disease in this patients’ population. It had no effects on MDA, total antioxidant status, total cholesterol, triglyceride, high-density lipoprotein cholesterol, and low-density lipoprotein cholesterol [111].

Contrarily, Chang and colleagues did not observe significant results in CRP levels by 600 mg of ALA supplementation for 8 weeks in diabetic HD patients [112].

Similarly other two clinical trials, examining the anti-oxidant effects of ALA combined with mixed tocopherols in patients with CKD in both conservative and dialysis treatment, failed to give significant positive results [113, 114].

More recently, lipoic acid demonstrated positive effects in the treatment of diabetic nephropathy [115–118]. In particular, it was able to prevent renal insufficiency, glomerular mesangial matrix expansion, and glomerulosclerosis by restoring glutathione and reducing malondialdehyde levels [119].

We believe that such differences in outcomes might be primarily attributed to differences in genetic characteristics of patients and, in most studies, by limitations such as blind administration of study interventions, small sample size, and short period of follow-up. To avoid these strong biases, nephrological research community should undertake well organized multicenter international clinical trials.

### Omega 3 polyunsaturated fatty acids (Omega-3 PUFAs)

Omega-3 are a family of polyunsaturated fatty acids (PUFAs) (including eicosapentanoic acid (EPA), docosapentaenoic acid (DPA) and docosahexanoic acid (DHA)) that play a major role in modulating the structure and function of cell and organelle membranes [120, 121]. A major source of these substances is the fish (mainly fish oil) [122].

They exert anti-inflammatory functions by reducing the expression/production of adhesion molecules, chemotactic factors, pro-inflammatory cytokines (TNF-a, IL-1b and IL-6) [123–125]. These effects are mainly due to the suppression of the IkB phosphorylation with a subsequent NFkB inactivation [126].

Moreover, being precursor of prostaglandins (I3 and E3), prostacyclins, thromboxanes A3 and leucotrienes B5, they have anti-thrombotic effects [127–129].

They also participate in membrane fluidity, ion channels transport (sodium, potassium and calcium) and mitochondrial biogenesis [130–132].

Additionally, they are also known to have anti-oxidant properties. They enhance endogenous antioxidant defense systems such as GSH through increased activity of ɣ-glutamyl-cysteinyl ligase, glutathione reductase and glutathione S-transferase [133]. DHA and EPA are incorporated into the phospholipid bilayer of cells where they displace arachidonic acid and reduce the ROS production by COX2 and xanthine oxidase pro-oxidant pathway [134].

From a clinical point of view, Omega-3 supplementation may have beneficial effects on lipid profile [135–137], blood pressure maintenance and redox status [138–141], together with important cardioprotective properties [142–144].

Additionally, some studies reported that fish oil therapy significantly reduced diastolic (7 to 15 mmHg) and/or systolic blood pressure (16 to 30 mmHg) [145–147] in dialysis patients. However contrasting results have been published [148–150].

Several evidences suggest that thiobarbituric acid reactive substances (TBARS) level are reduced and SOD, glutathione peroxidase and catalase (CAT) activities increased after initiating treatment with Omega-3 [151]. Interestingly it has been demonstrated their ability to reduce 5- lipoxygenase activity, an enzyme responsible for apoptosis in PBMC of end stage renal disease patients [152, 153].

These findings were also confirmed in a study performed in a rat model of CKD supplemented with omega-3 for 12 weeks showed downregulation of prooxidant, proinflammatory and profibrotic pathways [154].

The long nephrology story of Omega-3 and these recently published studies in HD patients raised new hopes and, according to their authors, should promote randomized clinical trials with fish oil supplementation to improve cardiovascular outcomes in this setting.

However, as clearly suggested by Teta [155] the lessons learned from studies in non-dialysis settings, coupled with the consistent history of negative trials in the dialysis population, should invite caution. Further steps may be required before investment of resources in a randomized clinical trial with Omega-3 in this population. Additional epidemiological evidence from larger samples of HD patients should be undertaken and interventional trials should be performed to define the best dose for each patient to reach sufficient blood levels of omega-3 fatty and to avoid complications (e.g., risk of bleeding especially in patients taking aspirin, clopidogrel, and anticoagulants, which are prevalent in CKD and cardiovascular disease population).

### Vitamin E

Vitamin E indicates a group of 8 structurally related compounds (comprising α, β, ɣ, δ tocopherol and the corresponding tocotrienols) [156]. However, α-tocopherol having higher bioavailability *in vivo*, has been more extensively studied.

Vitamin E has been shown to regulate superoxide generation in human neutrophils and monocytes and mitochondrial ROS in skeletal muscle and liver [157–159]. This protective effect on oxidative stress attenuates the onset and development of several cardiovascular disease, aging and other chronic/degenerative diseases (including CKD).

Additionally, this vitamin has also been shown to mediate the activation and gene expression of protein kinase C [160–162], transcription factor activator protein-1 [160, 163], transforming growth factor beta-1 [164], NFkB and related transcription factors [160, 165]. These factors are known to play important roles in mediating a number of pathophysiologic events including platelet adhesion and aggression and mural thrombus [166, 167], vascular smooth muscle cell proliferation [160, 168], apotosis [169] and glomerulosclerosis [170].

Therefore, dietary vitamin E intake by regulating the above mentioned biological/biochemical pathways and redox-sensitive biologic machineries could prevent or delay the progression of chronic systemic alterations (including chronic kidney damages) [159]. These protective effects have been also described in dialysis treated CKD patients [171, 172].

The SPACE study tested the cardiovascular preventive effects of vitamin E supplementation (administered at high-dosage, 800 IU/day) in HD patients with previous cardiovascular events. During the long follow-up (519 days) they found 40 % reduction in both composite cardiovascular-events and myocardial infarction (70 %) [173].

However, the beneficial effects of this agent in patients with renal damage is largely debated since a meta-analysis showed an increased mortality for all causes in patients affected by CKD treated with a daily dose > 400 IU [174].

Vitamin E seems also useful when bonded to dialysis membranes. In fact, in several studies oxidative stress and inflammatory markers were reduced together with an improvement in hemoglobin level and a reduction in Erythropoiesis-Stimulating Agents (ESA) requirement by long-term use of dialysis filters coated with vitamin E [175–179].

It is unquestionable that the results of the above mentioned studies suggest that vitamin E supplementation may be an effective accessory therapy to combat oxidative stress-lowering lipid peroxidation in CKD and HD patients. However, the correct clinical use of this vitamin in nephrology need to be better clarified.

### Vitamin C

Vitamin C (ascorbic acid) is a water-soluble antioxidant found in some vegetables and fruits and distributed both in intra- and extracellular fluids. It scavenges ROS and reactive nitrogen species by forming semidehydroascorbic acid and may thereby prevent oxidative damage to important biological macromolecules [180].

It has been demonstrated that patients with CKD show a reduction in both the total Vitamin C concentration and the active form (ascorbate) probably caused by a diminished intake of fruits and vegetables in order to avoid iperkaliemia and the loss during HD treatment [181–183]. Another possible explanation could be an impairment of enzymatic or non-enzymatic recycling of ascorbate from dehydroascorbate (the oxidized form of vitamin C), since the recycling is largely GSH dependent [184] and dialysis patients have a marked GSH deficiency [182].

To avoid this condition, currently, oral ascorbate/week (1–1.5 g), or parenteral ascorbate/dialysis session (300 mg), are recommended to compensate for subclinical deficiency, although evidence for such recommendations is scarce [183].

In a cross-over study, patients treated for three months with 200 mg/day vitamin C showed decreased CRP level and augmented prealbumin concentration [185].

Additionally, 2 months treatment with intravenous vitamin C in dialyzed patients using vitamin E-coated membranes significantly reduced oxidative stress, avoided the reduction of erythrocyte reductases activity and decreased the level of pro-inflammatory cytokines [179].

Ascorbic acid has also been used to improve response to ESA [186] through an increase in hemoglobin concentration and transferrin saturation. Use of ascorbic acid may enhance iron availability through 2 mechanisms: as a reducing agent that can mobilize iron from its storage sites and through its role of integration of iron into the heme moiety [187, 188].

Vitamin C has, also, anti-apoptotic effects by maintaining the mitochondrial membrane potential and protecting mtDNA from oxidant insults [189–192].

Although interesting, the use of this agent remains unusual in nephrology.

## Plant extracts with antioxidant properties

### Nigella sativa

Nigella sativa (or black cumin) is a herbaceous plant growing particularly in Mediterrean area and in India [193] largely employed for culinary and medicinal purposes (treatment of pulmonary disorders, cardiovascular diseases, fever and influenza) [194]. Biological effects of Nigella sativa seeds seem to be related to their oil components.

Its seed oil contains an elevated quantity of polyphenols and tocopherols [195]. Thymoquinone (TQ) and its derivatives (dithymoquinone, thymohydroquinone, and thymol) [196] are the most abundant.

Their quinine structure confers to these molecules a significant antioxidant activity as scavenger of superoxide, hydroxyl radical and singlet molecular oxygen [197–199]. Additionally, TQ has anti-inflammatory property by inhibiting ecoisanoid, thromboxane B2 and leukotrienes B4 [200].

In medicine, TQ and its derivatives are tested as mitochondria-targeted antioxidants [201] and Nigella Sativa has been employed as preventive agent against doxorubicin (DOX), gentamicin, vancomycin and cisplatin nephro-toxicity [202, 203] by increasing glutathione peroxidase activity [204, 205] and against nephrotic syndrome-associated clinical complications [206].

As demonstrated by Badary et al. in a DOX-induced hyperlipidemic nephropathy rat model, treatment with TQ produced a significant reduction of nephritic syndrome-related clinical signs and complications (massive albuminuria, proteinuria, hyperlipidemia, hypoalbuminemia and hypoproteinemia). These signs resemble histologically and clinically focal and segmental glomerulosclerosis, one of the cause of CKD [206, 207]. The possible molecular mechanism for these positive effects could be a reduction of oxidative stress. In fact, although the exact molecular mechanism mediating the DOX-induced nephropathy remains unknown, it is believed that the toxicity may be mediated by ROS which cause glomerular injury and increased glomerular capillary permeability [208].

Cellular models have, also, clearly demonstrated that Nigella Sativa may have dose-related antiproliferative and cytotoxic effects [209–211].

According to these few published data, it is plausible that this agent may represent in future a new valid therapeutic tool in clinical nephrology, but at the moment, the absence of strong clinical evidences suggests a prudence in its employment in the treatment of patients with chronic renal disease.

### Curcumin

Curcuma is a traditional Asian spice derived from the homonymous rhizome of the ginger family (Zingiberacee).

It has been used in traditional Asian medicine (Ayurveda, Chinese, Arabian) for centuries. Recently a great number of studies have demonstrated that curcumin, the mean curcuminoid contained in Curcuma longa, exhibits high anti-inflammatory and antibacterial properties [212–216].

Additionally, this agent can modulate several enzymes, cytokines, transcription factors, growth factors, receptors, micro RNA (miRNA) [217–219] that determine its dual antioxidant activity [220]. In detail, Curcumin is able to directly scavenge superoxide anion, hydroxyl radicals, H_2_O_2_, singlet oxygen, nitric oxide, peroxynitrite [221–225] and peroxyl radicals probably by means of phenolic groups in its molecular structure [222].

Curcumin has also indirect antioxidant ability mediated by the induction of the expression of cytoprotective proteins such as SOD, CAT [218], glutathione reductase (GR), glutathione peroxidase (GPx) [226], heme oxygenase 1 (HO-1) [227], glutathione-*S*-transferase (GST), NAD(P)H: quinone oxidoreductase 1 [228] and γ-glutamylcysteine ligase [229].

The potential therapeutic effects of Curcumin have been evaluated in several animal models of renal diseases [230–238] and clinical trials for cancer, Alzheimer’s disease, ulcerative colitis, diabetes [217, 239].

Khajehdehi et al. have shown that, oral supplementation of turmeric/curcumin (one capsule with each meal containing 500 mg turmeric, of which 22.1 mg was the active ingredient curcumin-3 capsules daily for 2 months) has strong protective renal effects (reduction of proteinuria and inflammatory background) in patients with overt type-2 diabetic nephropathy together with a decrease in systolic blood pressure in patients suffering from relapsing or refractory lupus nephritis indicating a direct podocyte effect and making it a promising remedy for chronic glomerulonephritis and CKD [240, 241].

Finally, curcumin was able to induce a cardiovascular protection against CKD-associated cardiac remodeling, in part due to a preservation of the mitochondrial function [242].

However, at the moment, all these promising clinical evidences are not so sufficient to start a large utilization of this compound in our patients, but in future we believe that Curcumin could be employed in selected and well defined patients affected by glomerular pathologies and chronic renal impairment.

### Quercetin

Quercetin (IUPAC nomenclature: 3, 3′,4′,5,7-pentahydroxyflavanone) is a flavonol presents in several aliments (e.g., onions, shallots, apples, berries, grapes, cappers, brassica vegetables, tea, red wine) [243] with strong anti-oxidant properties including scavenging of free radicals, inhibition of xanthine oxidase and decrement of lipid peroxidation [244–247]. Additionally, it has anti-inflammatory effects by suppressing the MAPK and NFkB signal transduction pathways [248], by modulating NOS and COX-2 synthesis and down-regulating CRP [249–251].

The renoprotective effect of this substance has been assessed in several models of toxic injury [252–254] and Shoskes et al. have shown that quercetin prevent renal injury in rodent models of ischemia/reperfusion and ureteral obstruction [255–257].

Interestingly the immune modulator effect of quercetin seems to be mediated also through two mechanisms: inhibition of the lymphocytes proliferation by arresting cell cycle in G1/S phase [258] and down-regulation of IL-2 synthesis [259, 260]. These findings led to an open label phase I study in renal transplant recipients taking Oxy-Q which contains 400 mg of curcumin and 100 mg of quercetin [261]. In patients with poor renal function serum creatinine improved and in patients with delayed graft function, there was an enhancement in renal function.

Subsequently the same authors performed a randomized placebo controlled study with Oxy-Q started after renal transplantation and taken for 1 month. Patients were randomized into three groups: control (placebo), low dose (one capsule, one placebo) and high dose (two capsules). The high dose bioflavonoid group had the lowest serum creatinine values, the least neurotoxicity and an acute rejection rate at 6 months (including subclinical rejection) of 0 % and the higher early graft function. Considering that urinary HO-1 was higher in bioflavonoid groups, authors suggested that these positive effects could be possible thanks to this enzyme induction [262]. HO-1 is a biological element able to reduce ischemia reperfusion damage and alloimmunity in renal transplant recipients [263]. However, authors conclude that these results were completely observational and the mechanism of the increased urinary HO-1 activity deserves further study [262].

Moreover, it has been suggested that Quercetin may also prevent tissue oxidative damages and attenuate renal damage in streptozotocin-induced diabetic rats [264–266].

As concern mitochondria Davis et al. reported that supplementation of quercetin for 7 days in mice induced PGC-1α and Sirtuin 1 (SIRT1) mRNA up-regulation and enhanced mtDNA and cytochrome c concentration [267]. Quercetin inhibits complex I ability to generate O_2_^−^ [268].

It is also used as medication to treat cancer, cardio-vascular diseases, systemic inflammation and gastrointestinal pathologies [269].

### Resveratrol

Resveratrol (3,5,4′-trihydroxystilbene) is a natural phenol, contained in red wine and plants such as grapes, peanuts and berries [270]. It has antioxidant, anti-inflammatory, anti-mutagenic and anticancer properties [271–274].

This phytochemical exerts antioxidant effect by scavenging ROS directly and inducing the expression of several antioxidant enzymes such as SOD, CAT through NRF-2 [275].

Resveratrol activates the axis AMPK/SIRT-1/PGC-1α [276], and attenuates aldosterone-induced mitochondrial dysfunction and podocyte injury [277].

Resveratrol inhibited both 5-lipooxygenase (LOX) and cyclooxygenase (COX) activities resulting in a reduced accumulation of inflammatory mediators [278].

Thanks to its antioxidant mechanisms and influencing MAPK and TGF-β1/Smad3 pathways, Resveratrol prevents epithelial to mesenchymal transition and renal fibrosis [279–284].

It has also been shown that this phenol may antagonize acute kidney injury due to cisplatin, ischemia-reperfusion and sepsis in animal models [285–287].

Unfortunately, resveratrol has poor bioavailability making difficult to translate the aforementioned *in vitro* findings into clinical trials [288]. At the moment several clinical trials on resveratrol are ongoing involving several metabolic and inflammatory systemic diseases.

### Green tea polyphenols

The major polyphenols present in green tea are epigallocatechin 3-O-gallate, epicatechin 3-O-gallate, epigallocatechin and epicatechin. Beneficial actions of catechins are mostly due to the antioxidant properties, to the ability to chelate metal ions such as copper (II) and iron (III) and to form stable semiquinone free radicals [289–293]. The epigallocatechin-3-O-gallate (EGCG) is the most abundant and most active in green tea [294].

Several mechanisms have been linked to the anti-inflammatory property of EGCG such as: a) Inhibition of NFkB [295] and b) activation of AMPK that inhibit the production of several proinflammatory mediators including TNF-α, IL-1β, IL-6, monocyte chemoattractant protein-1, inducible nitric oxide synthase (iNOS) and cyclooxygenase-2 with LPS stimulation [296–298]. More recently Qin et al. have also reported a direct interaction between EGCG and chemokines with a consequent limitation of their biological effects [299].

EGCG prevented the induction of vascular adhesion molecule-1 by TNF α and IL-1, which subsequently reduced monocyte adhesion [300].

In rats subjected to unilateral ureteral obstruction, EGCG administration caused up-regulation and nuclear translocation of NRF2 with consequent enhancement of antioxidant enzymes such as glutathione peroxidase, glutathione S-transferase, γ-GCS and HO-1 [301].

At the same time EGCG alleviates glomerular and tubular injury and attenuates renal interstitial fibrosis through TGF-β/Smad signaling pathway inhibition and NFkB upregulation [302, 303].

In Wistar rats subjected to ischemia-reperfusion renal damage together with LPS injection, the administration of EGCG reduces the activity of myeloperoxidase and protects kidney from peroxynitrite-induced damage [304].

Green tea polyphenols (daily dose, 400 mg) administered for 6 months to 50 patients on dialysis decreased the blood levels of methylguanidine [305] an uremic toxin that accumulates with the progression of renal failure. Furthermore, the same authors reported beneficial effects on renal function with green tea polyphenols administration to nephrectomized rats [306].

Also during diabetic nephropathy, EGCG leads to improvement of proteinuria, reduction of advanced glycosylation end products (AGE), hyperglycemia, lipid peroxidation thanks to its antioxidant activity and inhibition of NFkB [307].

Moreover, these substances may protect kidneys by several drugs such as ciclosporin, cisplatin, gentamicin through their anti-oxidative properties [308–310].

## Conventional drugs with antioxidant “side effects”

### N-acetyl cysteine (NAC)

N-acetylcysteine is a modified form of the amino acid cysteine, in which the nitrogen atom of the amino group is attached to an acetyl group. It has received attention because of its antioxidant capacity primarily due to its ability to drive the synthesis of the powerful antioxidant GSH [311]. Moreover NAC reacts fast and directly with radical ^·^OH, radical ^·^NO2, CO3^·-^ [312].

Upon deacetylation, NAC becomes L-cysteine, entering cells where it may serve as a precursor for GSH synthesis. In kidney subjected to ischemia/reperfusion injury GSH level is reduced and can be restored by NAC [313].

In cultured human proximal tubular epithelial cells, NAC reduced lipid peroxidation and maintained the mitochondrial membrane potential, thereby preventing apoptosis following H2O2 administration [314].

NAC has an important vasodilatory effect maybe mediated by its ability to stabilize nitric oxide or by inhibiting angiotensin-converting enzyme [315, 316].

Since vasoconstriction is believed to be a pathogenic factor in contrast-induced nephropathy, vasodilatory effects may prove helpful and NAC has been the subject of numerous trials with mixed results [317–320]. The great difference in the clinical trials is the degree of risk of the patients involved. It seems to have positive effect on patients with renal dysfunction [321]. Another important point is the route of administration [322]. Given into account that NAC has an indirect effect, by acting on GSH metabolism, early dosing may be necessary [323]. Overall a positive effect of NAC on contrast-induced nephropathy is too far to come but since it is safe and well tolerated, intravenous NAC as a prophylactic agent for prevention of contrast-induced nephropathy is adequate [323].

Additionally, even if controversial, some studies showed beneficial effects of NAC supplementation in both PD and HD patients, such as increment in GFR, urine volume, reduction of IL-6 and MDA level and composite cardiovascular endpoints [324–328].

Interestingly, Tepel et al. conducted a prospective, randomized, placebo-controlled trial in 134 HD patients randomly assigned either to receive acetylcysteine (600 mg BID) or placebo. The primary end point was a composite variable consisting of cardiac events including fatal and nonfatal myocardial infarction, cardiovascular disease death, need for coronary angioplasty or coronary bypass surgery, ischemic stroke, peripheral vascular disease with amputation, or need for angioplasty. Secondary end points included each of the component outcomes, total mortality, and cardiovascular mortality. In the acetylcysteine group 28 % patients had a primary end point while 47 % of the control group (*p* = 0.03). No significant differences in secondary end points or total mortality were detected [327].

Contrarily, several studies reported no effect of NAC supplementation neither on surrogate markers of cardiovascular injury nor on kidney function in patients with CKD [329–331].

The exact reasons for these negative results are not completely known, but they could be due to the relatively short treatment period and to a not standardized treatment dose. In fact, other authors reported that NAC has some value as an antioxidant, but only in certain conditions [332]. Moreover, at doses as low as 1200 mg daily, NAC may even exert pro-oxidative properties in people with normal intracellular GSH level [333]. However, these studies underline the necessity to undergo large multicenter trails to better define the therapeutic effect of NAC supplementation in CKD patients.

### Carvedilol and captopril

Besides conventional use of carvedilol and captopril in the treatment of cardiovascular disease, these drugs explicate a potent antioxidant and anti-apoptotic activities.

Carvedilol is a nonselective beta-blocker, antagonizing β-1 and β-2 receptors with antioxidant properties attributed to the presence of a carbazole moiety in the molecule [334–336].

Several studies have reported protective effect of carvedilol against ischemia/reperfusion and drugs-induced nephrotoxicity [337–341].

Captopril is an angiotensin-converting enzyme (ACE) inhibitors with antioxidant properties due to a thiol group in its structure that has both the ability to scavenge free radical directly and to enhance antioxidant enzyme level [342, 343].

Experiments performed on a mouse model of acute kidney injury induced by ischemia/reperfusion demonstrated that captopril (an anti-hypertensive drug) determined important renal positive effects by inhibiting angiotensin-II activity and reducing parenchymal inflammation.

However, in the last phase of reperfusion, captopril was no longer effective [344].

### Allopurinol

Allopurinol is a xanthine oxidase (XO) inhibitor used worldwide to treat hyperuricemia. XO is an enzyme that catalyzes the conversion of hypoxanthine to xanthine and finally to uric acid together with the production of ROS.

Uric acid is increased in CKD patients and is emerging as a potentially modifiable risk factor for CKD. The increment in uric acid results in oxidative stress and endothelial dysfunction with consequent development of systemic and glomerular hypertension in association with elevated renal vascular resistance and reduced renal blood flow [345–347]. Hyperuricemia was also able to induce an epithelial-to-mesenchymal transition, with direct effects on the tubular cell population [348].

There are several mechanisms mediating these effects: uric acid stimulates vascular smooth muscle cell proliferation with the activation of mitogen-activated protein kinases (MAPK) [349, 350], growth factors (PDGF), chemokines (monocyte chemoattractant protein-1 [MCP-1]), and inflammatory enzymes (COX-2) [351]. On endothelial cells, uric acid activates the renin-angiotensin system with augmented apoptosis and vascular dysfunction [352, 353].

Moreover long-term hyperuricemia induces hypertension, renal vasoconstriction, tubular damage, renal cortex oxidative stress, and mitochondrial impairment shown by oxidative phosphorylation uncoupling, reduced ATP renal content and lower mitochondrial DNA [354]. Treatment with allopurinol prevented these alterations [355].

In humans, several trials have reported beneficial effect of treatment with allopurinol on progression of kidney disease and cardiovascular events [356–361].

## Mitochondria-targeted molecules

Although interesting, the conventional antioxidants are still far from a practical clinical employment. In fact, a great limitation of these antioxidants is their inability to reach *in vivo* an adequate mitochondrial concentration [362]. Therefore, in the last years, a great number of research strategies have been developed to minimize this condition.

Firstly, most of these molecules were synthesized by conjugating well known antioxidants with the lypophylic triphenylphosphonium (TPP) cation that enables such compounds to move rapidly through biological membranes and, because of its positive charge, to drive them inside mitochondria [363].

The first produced mitochondria-targeted antioxidant was MitoE, which comprises the α-tocopherol moiety of vitamin E conjugated to TPP by a two carbon chain [364]. MitoE is taken up rapidly by mitochondria making this molecule more effective than the untargeted α-tocopherol to prevent lipid peroxidation.

With the same research and technical approach it has been synthetized MitoQ, a quinone moiety linked to TPP by a 10-carbon alkyl chain [365–367]. MitoQ accumulation within mitochondria is driven by the membrane potential and it is absorbed to the matrix surface of the inner membrane where it exerts its protective effects against lipid peroxidation [366].

In animal models, MitoQ has been employed in several studies aimed to verify its protective effects against diseases involving mitochondrial oxidative damage (e.g., cardiac ischemia/reperfusion injury [368], endothelial damage induced by hypertension [369]).

MitoQ added to the cold storage fluid used to preserve the organ before kidney transplantation, prevented mitochondrial dysfunction, improved cell viability and renal morphology [370]. Likewise mitoQ intravenously administered to mice 15 min prior to occlude the renal vessels exerts protective effects on renal function and against oxidative damage [371, 372].

Also in a mouse model of diabetic nephropathy this compound demonstrated an important anti-fibrotic activity and a defensive effect against chronic glomerular damage [373].

From a clinical point of view, MitoQ (generally administered at oral dosing of 1 mg/kg) has undergone phase I and II clinical trials [374, 375].

Another interesting mitochondria-targeted molecule is MitoSOD, designed by attaching TPP to the pyridine ring of M40403 (a non-peptidyl mimetic of MnSOD). This agent accumulates into mitochondria and it protects against oxidative damage induced by O_2_^−^ [376, 377]. At our knowledge, no clinical trials using this molecule are ongoing.

Mito-TEMPO, then, a nitroxide linked to TPP, with similar effects to MitoSOD, has positive effects in hypertension-related vascular injury by reducing the O_2_^-^ and increasing the bioavailability of nitric oxide with subsequent endothelial-dependent relaxation [378].

In addition, MitoTempo (10 mg/kg) given at 6 h post cecal ligation and puncture (CLP) in a murine model of sepsis demonstrated reversed mitochondrial impairment together with an improvement in renal microcirculation and glomerular filtration rate [379].

In order to ameliorate the mitochondrial up-take of targeted molecules, in the last years, researchers are introducing new agents such as Szeto-Schiller (SS) peptides, promising molecules constituted by alternating aromatic residues and basic amino acids that have some features rendering them potent antioxidants: (1) they are taken up into cells in an energy-independent nonsaturable manner, (2) have a sequence motif that targets them to mitochondria, (3) are very potent in reducing intracellular ROS and preventing cell death at very low concentration [380–382] and (4) their uptake is not dependent on mitochondrial membrane potential. The antioxidant ability seems due to a tyrosine or 2,6-dimethyl-L-tyrosine (Dmt) residues and to their position in the sequence [381, 383].

These peptides have been tested in several animal models of oxidative damage such as myocardial infarction, ischemia reperfusion, amyotrophic lateral sclerosis, and pancreatic islet cell transplantation [384–388]. In a rat model of CKD performed by unilateral ureteral obstruction, SS-31 (1 or 3 mg/kg) given 1 day before and throughout the 14 days of obstruction, significantly decreased tubular apoptosis, macrophage infiltration, fibrosis and it increased tubular proliferation [389].

In a rat model of ischemia/reperfusion SS-31 was administered subcutaneously 30 min before a 30 or 45 min long bilateral occlusion of renal blood flow, at the onset of reperfusion and 2 h later. It preserved renal tubular architecture, reduced apoptosis and maintained mitochondrial integrity and function with full recovery of ATP content after reperfusion. As a consequence, oxidative stress and inflammation were reduced and tubular cell regeneration was accelerated [390].

Interestingly, Liu et al. have demonstrated protective properties of SS-31 during ischemia/reperfusion also in capillary endothelial cells. As reported in tubular epithelial cells, SS-31 protects mitochondrial structure and prevents endothelial cell swelling, cell detachment and cell death [391]. The mechanism seems to be mediated by an interaction between SS-31 and cardiolipin, an anionic phospholipid expressed in the inner mitochondrial membrane. This interaction prevents cardiolipin peroxidation by inhibiting cytocrome c peroxidase activity [392].

SS-31 is now under evaluation in a multinational clinical trial for reperfusion injury in patients with acute coronary events (NCT01572909), and in a Phase 2 trial to assess the effectiveness on improving renal function after angioplasty for severe renal artery stenosis (NCT01755858).

Then, on the basis of SS-31, more recently Cerrato and coworkers have synthesized novel peptides called mitochondrial cell-penetrating peptides (mt-CPPs). CPP are short, nontoxic peptides with amphypathic and cationic properties able to cross the cellular membrane [393]. Mt-CCP-1 is not toxic even at high concentration, did not perturb Δψm and, interestingly, its amount into the cells is higher than SS-31 [393].

There are numerous CPPs available with different sequence and physicochemical properties that can be conjugated with different cargoes (small drugs, peptide or larger cargoes such as oligonucleotide, proteins and plasmids) very useful to deliver drugs into the target of interest [394].

## Conclusions

Emerging evidences suggest that dysfunctional mitochondria have a primary role in the development of CKD as well as in comorbidities related to CKD and underline their role as new therapeutic targets.

A variety of agents (including endogenous and food derived antioxidants, natural plants extracts, mitochondria-targeted molecules) combined with conventional therapies and an appropriate life style could help clinicians to reach this objective. However, for a correct utilization of these agents is extremely important to understand their effects and to identify the correct target of interventions. In fact, although the beneficial effects of these compounds are well known, large clinical trials are needed to provide more definitive information on their efficacy in CKD.

Finally, future strategies (including pharmacogenomics [395]) should be undertaken to identify target patients potentially responsive to mitochondria-related anti-oxidant treatments.

## Abbreviations

CKD: Chronic kidney disease
HD: Hemodialysis
PD: Peritoneal dialysis
RRT: Renal replacement therapies
ROS: Reactive oxygen species
PBMC: Peripheral blood mononuclear cells
GDP: Glucose degradation products
OXPHOS: Oxidative phosphorylation system
NADH: Nicotinamide adenine dinucleotide
FADH2: Flavin adenine dinucleotide
PGC-1α: Peroxisome proliferator-activated receptor c coactivator 1
NRF1: Nuclear respiratory factor 1
TFAM: Mitochondrial transcription factor A
COX6C: Cytochrome c oxidase subunit VIc
COX7C: Cytochrome c oxidase subunit VIIc
UQCRH: Ubiquinol-cytochrome c reductase hinge protein
MCAD: Medium-chain acyl-CoA dehydrogenase
SOD2: Superoxide dismutase 2
GSH: Glutathione
JNK: Jun-N-terminal Kinase
CoQ10: Coenzyme Q10
PTP: Permeability transition pore
NFkB: Nuclear factor kappa B
ALA: Alpha-lipoic acid
VSMC: Vascular smooth muscle cells
MDA: Malondialdehyde
PUFA: Polyunsaturated fatty acids
EPA: Eicosapentanoic acid
DPA: Docosapentaenoic acid
DHA: Docosahexanoic acid
TBARS: Thiobarbituric acid reactive substances
ESA: Erythropoiesis-Stimulating Agents
CRP: C-reactive protein
TQ: Thymoquinone
NRF2: Nuclear factor (erythroid-derived 2)-like 2
CAT: Catalase
NOS: Nitric oxide synthase
COX2: Cyclooxygenase-2
AMPK: 5′ AMP-activated protein kinase
SIRT1: Sirtuin 1
LOX: 5-lipooxygenase
TGF-β: Transforming growth factor beta
EMT: Epithelial to mesenchymal transition
LPS: Lipopolysaccharide
EGCG: Epigallocatechin-3-gallate
MG: Methylguanidine
NAC: N-acetyl cysteine
ACE: Angiotensin-converting enzyme
α-SMA: Alpha smooth muscle actin
SS: Szeto-Schiller
